# Reproductive surgery remains an essential element of reproductive medicine

**DOI:** 10.52054/FVVO.16.2.022

**Published:** 2024-06-28

**Authors:** B Urman, B Ata, V Gomel

**Affiliations:** Dept. of Obstetrics and Gynecology, Koç University School of Medicine, Istanbul, Turkey; Women’s Health and Assisted Reproduction Center, American Hospital of Istanbul, Turkey; ART Fertility Clinics, Dubai, United Arab Emirates; Dept. of Obstetrics and Gynecology, University of British Columbia, BC, Canada

**Keywords:** Reproductive surgery, tubal reconstructive surgery, laparoscopy, hysteroscopy, fibroids, endometriosis

## Abstract

**Background:**

Reproductive surgery has long been neglected and is perceived to be simple surgery that can be undertaken by all gynaecologists. However, given the ever-expanding knowledge in the field, reproductive surgery now comprises surgical interventions on female reproductive organs that need to be carefully planned and executed with consideration given to the individuals symptoms, function of the organ and fertility concerns.

**Objectives:**

To discuss the different perspectives of reproductive surgeons and other gynaecological surgeons, e.g., gynaecological oncologists, and advanced minimally invasive surgeons, regarding diagnosis and management of pelvic pathology that affects reproductive potential. Furthermore, to highlight the gaps in knowledge and numerous controversies surrounding reproductive surgery, while summarising the current opinion on management

**Materials and Methods:**

Narrative review based on literature and the cumulative experience of the authors.

**Main outcome measures:**

The paper does not address specific research questions.

**Conclusions:**

Reproductive surgery encompasses all reproductive organs with the aim of alleviating symptoms whilst restoring and preserving function with careful consideration given to alternatives such as expectant management, medical treatments, and assisted reproductive techniques. It necessitates utmost technical expertise and sufficient knowledge of the female genital anatomy and physiology, together with a thorough understanding of and respect to ovarian reserve, tubal function, and integrity of the uterine anatomy, as well as an up-to-date knowledge of alternatives, mainly assisted reproductive technology.

**What is new?:**

A holistic approach to infertile women is only possible by focusing on the field of reproductive medicine and surgery, which is unattainable while practicing in multiple fields.

## Introduction

Reproductive surgery entails a vast array of surgical procedures performed with the aims of correcting deformity or excision of pathology while improving or preserving the future reproductive potential of the patient. Trauma caused by non- indicated or less than optimal procedures may result in irreparable damage in terms of adhesions (intraperitoneal or intrauterine), tubal occlusions, loss of myometrial tissue or severe insult to the ovarian reserve (Gomel et al., 1996; Ruiz-Flores and Garcia-Velasco, 2012; Gomel and Koninckx, 2016; Yilmaz Hanege et al., 2019). Gains by complete and immediate resection of a disease, e.g., endometriosis, should be balanced against losses due to irreversible diminution of organ function (Etic Endometriosis Treatment Italian Club, 2019). Alternatives to surgery, i.e., medical treatment and assisted reproductive technology (ART), should be considered and discussed with the patient who is currently or may in the future contemplate starting a family (Gomel, 2015). On the other hand, ART alone must not be regarded as a panacea for all fertility problems, and it should be acknowledged that some patients benefit from reproductive surgery alone to enhance fertility or sometimes to maximise ART outcomes.

## Methods

This is a narrative literature review on fertility-promoting surgical procedures and includes the authors’ opinions informed by the literature as well as their personal experiences. As such, it does not address specific research questions but aims to highlight nuances in decision-making and management of surgical pathology that affect the reproductive potential of women. We aim to highlight differences between the perspectives of reproductive surgeons and non-reproductive gynaecological surgeons, e.g., gynaecological oncologists, and advanced minimally invasive surgeons.

### What is reproductive surgery?

Although this question seems to be quite straightforward it doesn’t have an easy answer as most surgical procedures undertaken by gynaecologists may fall into this category depending upon how they are viewed. In the 1960s and 70s, the term reproductive surgery was coined for procedures such as salpingostomy for hydrosalpinx and adhesiolysis for periadnexal adhesions and were largely undertaken by laparotomy without sufficient knowledge and understanding of de novo and secondary adhesion formation and peritoneal healing mechanisms (Gomel, 2005; Gomel and Koninckx, 2016). The aim of surgery was primarily to open a blocked tube enabling conception (Gomel, 1978; Rock et al., 1978). Other surgical interventions that may be viewed as reproductive surgeries such as myomectomy, ovarian surgery, surgery for tubal pregnancy, and endometriosis were similarly performed via laparotomy which often resulted in extensive postoperative adhesions limiting organ functions and thus nullifying originally intended benefits. Pioneers of microsurgery introduced the basic principles of delicate tissue handling under magnification with utmost respect to the surrounding normal tissues, constant irrigation to keep the peritoneal surfaces moist, and prevention of adhesion formation and reformation with a multitude of techniques based on pathophysiological principles of tissue healing (Gomel, 1980a; Swolin, 1993). The results were immediately obvious in terms of improved outcomes in conception rates (Gomel, 1978; Gomel, 1980a; Gomel, 1980b). Principles of delicate tissue handling and measures to prevent adhesion formation should be adapted to all gynaecological operations for immediate and long-term gains regarding postoperative pain, duration of hospitalisation, adhesion formation, preservation of fertility, and obstructive bowel complications.

The course of reproductive surgery gradually changed with the introduction and later widespread use of operative laparoscopy and subsequently with in vitro fertilisation (IVF) (Gomel, 1995; Gomel, 2019). Laparoscopy, initially performed for diagnostic purposes only, was rapidly recognised as a powerful tool for the treatment of pelvic pathology (Gomel, 1995). This evolution followed technological advances such as video imaging, better ancillary instruments, and improvement of energy deployment devices (Nezhat et al., 1992; Nezhat et al., 1994). The tenets of microsurgery and the importance of organ preservation, unfortunately, suffered during the transition period from laparotomy to laparoscopy (Gomel, 2019). This was further compounded by the ever-growing presence and success of IVF as a backup procedure that led to a more liberal approach of organ removal rather than preservation (Fan and Ma, 2016).

Different pathologies may be approached differently by different subspecialties within the specialty of gynaecology. This is no truer than in patients with reproductive disorders that require surgery and are counseled by reproductive endocrinologists, gynaecologic oncologists, or the minimally invasive gynaecologic surgeons. Subtle but important differences were noted in the way they counselled their patients, their preoperative workouts, and intraoperative and postoperative handling of the pathology (Petrozza et al., 2023). The differences resulted not from a lack of knowledge but from the patient desires, perceived priorities, and fertility concerns. Understandably, while gynaecologic oncologists adopted a more cancer-centred approach, reproductive surgeons appeared to be more focused on fertility issues.

Besides tubal reconstruction, reproductive surgery encompasses a wide array of surgical procedures performed for pelvic pathology with the aim of preservation or enhancement of fertility. These include surgery performed for congenital or acquired uterine anomalies, excision of ovarian cysts, endometriosis surgery, hysteroscopic surgery for myomas, polyps, intrauterine adhesions, or other intracavitary lesions (Bosteels et al., 2010). Principles and foundations laid by the pioneers of microsurgery are still valid today, be it for reproductive surgery performed by laparotomy, laparoscopy, or hysteroscopy.

### Fertility promoting surgery for pelvic adhesions and blocked tubes

**Figure 1:**
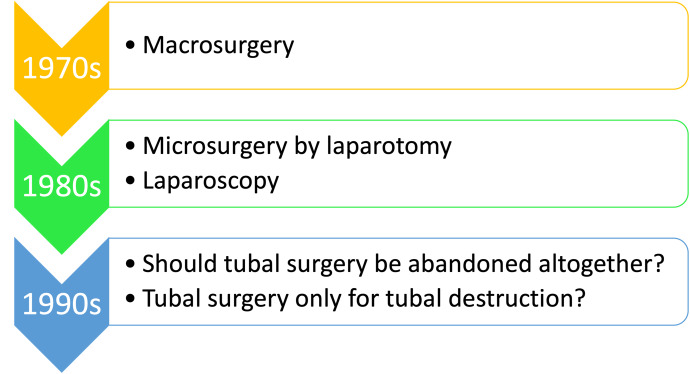
Evolution of fertility-promoting or tubal reconstructive surgery.

Fertility-promoting surgery for pelvic adhesions and blocked tubes, or in other terms tubal reconstructive surgery, has evolved over the years (Figure 1). While less delicate techniques such as macro surgery which can disrespect tissue was replaced by microsurgery, a time came when the discussion was centred on abandoning these procedures altogether. Fortunately, these difficult-to-acquire techniques have survived with the effort put forward by a few pioneers in reproductive surgery (Gomel, 2015).

**Figure 2:**
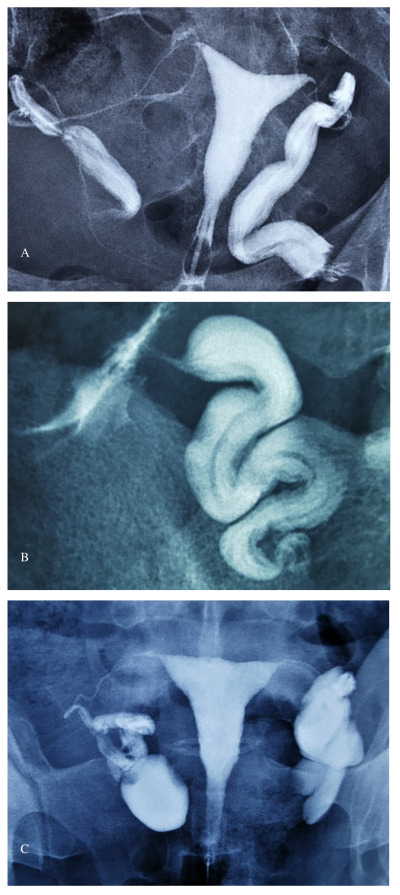
a: Hysterosalpingogram of a patient with bilateral hydrosalpinx who may benefit from salpingostomy (inner architecture of the tubes are well preserved); b. A phimotic fallopian tube with a well-preserved rugae. The patient may benefit from dilatation of the phimotic distal segment; c. Hys- terosalpingogram of a patient with bilateral hydrosalpinx who will most likely require a salpingectomy.

**Figure 3:**
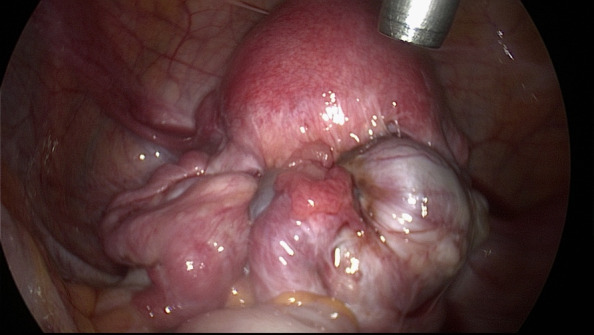
Patient with advanced-stage endometriosis. Both fimbriae appear to be normal despite severe adhesions resulting in a frozen pelvis.

The advantages of reproductive surgery training are obvious in cases of fertility-promoting surgery for women with pelvic adhesions and tubal occlusions. The reproductive surgeon who is adequately equipped with a detailed knowledge of tubal anatomy and physiology will be better prepared to deal with patients with reproductive issues (Munro and Gomel, 1994). Distal tubal occlusion due to different pathologies (i.e. infections, pelvic surgery, or endometriosis) should be dealt with different treatment algorithms (Marana et al., 1995; Milingos et al., 2000). A ‘one- size-fits-all’ approach of salpingectomy for all tubal pathologies should not be endorsed as some patients may benefit from reconstructive tubal surgery especially when pre and intraoperative imaging modalities show favourable findings and there are no additional infertility factors (Figure 2). This is especially true for patients with endometriosis as even severe forms of the disease may not affect the distal end of the tube and the fimbriae are usually spared (Figure 3). Classification of distal tubal disease based on findings at HSG and preoperative laparoscopy was proposed by Mage et al. (1986). The severity of tubal disease was defined by three factors: the appearance of mucosal folds at HSG, degree of distal occlusion, and appearance of the tubal wall at laparoscopy. While intrauterine pregnancy rates were over 50% in women with Grade 1 disease, none of the patients with Grade 4 disease conceived. Intrauterine pregnancy rates were likewise decreased in patients with increasing severity of pelvic adhesions. This study, like most other studies at that time, suffered from variable follow-up periods. A more recent retrospective case series of 434 women showed similar results after laparoscopic tubal reconstructive surgery (Audebert et al., 2014).

Salvaging the fallopian tube in women with endometriosis may prove to be extremely difficult due to severe pelvic adhesions as a consequence of the disease process itself and previous pelvic surgeries. Salpingectomy is usually preferred in women with previous pelvic infections that affect the inner architecture of the fallopian tube resulting in endosalpingeal damage (Figure 2c). Creating a stoma in such a tube will most likely result in reocclusion or an ectopic pregnancy at best. Intraoperative evaluation of the endosalpinx is possible with small diameter telescopes and should be used prior to deciding to remove or preserve a phimotic or blocked fallopian tube.

It is not rare that a patient will achieve a spontaneous pregnancy despite the presence of an unfavourable or hostile pelvic environment affected by infection, previous pelvic surgery or endometriosis. The liberal use of bilateral salpingectomy across the board will inevitably prevent spontaneous conceptions in the future which may occur even after multiple failed IVF attempts. When the fallopian tube/s are preserved careful postoperative assessment of their patency is required as reocclusion is a relatively common occurrence. This information should be clearly discussed with the patient.

Patients with phimotic or blocked tubes and multiple failed IVF attempts deserve special attention. The mechanism/s by which hydrosalpinx negatively affects embryo transfer outcomes are still unclear, but it may be due to factors such as mechanical flushing effect on the transferred embryos, decrease in endometrial receptivity, embryotoxic effects of the hydrosalpinx fluid and an inflammatory endometrial response (Strandell and Lindhard, 2002).

When blocked tubes are associated with fluid collection in the uterine cavity during ovarian stimulation or prior to embryo transfer the patient is best served by the removal of insulting tube/s (Pradervand et al., 2021). Effects of salpingectomy, tubal occlusion, and transvaginal aspiration of hydrosalpinx on IVF outcomes in women with tubal disease have been compared in multiple randomised controlled trials (Melo et al., 2020). While salpingectomy and tubal occlusion were found to significantly increase clinical pregnancy rates compared to no intervention, the benefit of transvaginal aspiration was deemed as uncertain (Melo et al., 2020). It is important to note that while hysteroscopic proximal tubal occlusion fared less effective than laparoscopic salpingectomy, laparoscopic tubal occlusion was similarly effective as salpingectomy (Melo et al., 2020). Likewise, hysteroscopic insertion of Essure device also performed worse than salpingectomy or laparoscopic proximal tubal ligation (Xu et al., 2017).

In women with prior tubal ligation, recanalisation of the tube (reversal of sterilisation) by mini-laparotomy or laparoscopic approaches (conventional or robotic) yields satisfactory pregnancy and live birth rates. Whether laparotomy or laparoscopy should be the preferred approach, however, has not been resolved due to lack of randomised controlled trials (George et al., 2013).

A direct comparison between IVF and sterilisation reversal is challenging due to inherent differences in reporting fertility outcomes. While surgical studies most often quote pregnancy rates over a predefined or retrospectively determined follow-up period, IVF results are reported as pregnancy or live birth rates per started cycle or per embryo transfer. Reversal of sterilisation may be the preferred approach in younger women and also older women with a normal male partner but failed IVF cycles. Clinical decision-making should include consideration of the risk of ectopic pregnancy, interval from sterilisation to reversal, type of sterilisation procedure, planned anastomotic site, and projected remaining tubal length (Garg and Milad, 2022). Lastly, the cost of surgery may be significantly less in countries where IVF is not funded by the health system.

Tubocornual anastomosis for proximal tubal occlusion is a largely abandoned procedure due to lower pregnancy rates and difficulty in performing the operation. Furthermore, there are only a few experienced microsurgeons who can perform this procedure.

Fertiloscopy or transvaginal hydro-laparoscopy championed by Gordts can be used in the evaluation of the infertile couple yielding a better understanding and delineation of pelvic anatomy and patency of the tubes (Gordts et al., 1998; Gordts et al., 2005). The technique has been found to be on par with laparoscopy for evaluation of infertility (Darai et al., 2000).

### Surgery for removal of myomas

Myomas are commonly encountered during the reproductive ages. There is much controversy regarding the optimal management and indications for surgery (Cook et al., 2010; Practice Committee of the American Society for Reproductive Medicine, 2017; Petrozza et al., 2023). Submucous fibroids, besides being a potential reason for infertility, also cause abnormal uterine bleeding and their removal is generally recommended in infertile women and women with recurrent pregnancy loss (Somigliana et al., 2007; De Angelis et al., 2022). It is unclear how myomas that do not distort the cavity adversely affect reproductive outcomes, however, they were shown to cause abnormal uterine peristalsis, and this was corrected after myomectomy (Yoshino et al., 2010; Yoshino et al., 2012). In a very recent meta-analysis, based on low to very low certainty of evidence from observational studies, 2-6 cm non- cavity-distorting intramural myomas were shown to decrease live birth rate in women undergoing IVF when compared with age-matched controls without myomas (Erden et al., 2023). However, whether myomectomy rectifies fertility is unclear as of today. Myomas distorting the uterine cavity, especially in patients with implantation failures and pregnancy loss will be probably best served by myomectomy (Practice Committee of the American Society for Reproductive Medicine, 2017).

**Figure 4:**
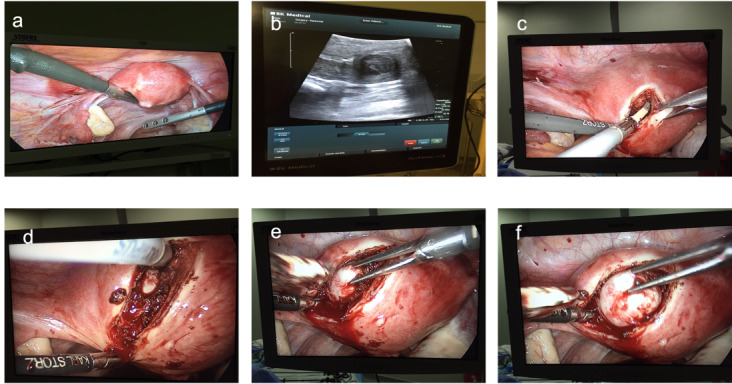
Deep intramural myoma indenting the uterine cavity removed laparoscopically under intraoperative ultrasound guidance: a. Locating the myoma with intraoperative ultrasound; b. Myoma visualized with intraoperative ultrasound; c. Placement of the serosal incision using the energy device; d. Identification of the cleavage plane of the myoma; e. Traction on the myoma using the endoscopic tenaculum; f. Removal of the myoma from the uterus.

Surgical removal should be contemplated after taking into consideration patients’ symptoms and careful anatomical mapping using ultrasound and if necessary, MRI. Consideration should be given to hysteroscopic evaluation and myometrial sampling if there is suspicion of a leiomyosarcoma (Bansal et al., 2008; Tantitamit et al., 2018). Increasing experience, and availability of various and better energy devices and surgical equipment rendered minimally invasive techniques feasible in most cases. Nonpalpable myomas can be identified using intraoperative endoscopic ultrasound thereby minimising the risk of incomplete surgery (Urman et al., 2018). This technique allows the removal of myomas that distort the uterine cavity but not the uterine serosa, hence cannot be visualised during laparoscopy/robotic surgery, both of which also have limited tactile feedback to help locate myomas (Figure 4). Transabdominal or transrectal ultrasound guidance can be used for hysteroscopic removal of type 3 myomas that do not encroach on the endometrial cavity. Myomectomy performed by inexperienced surgeons be it by laparotomy or laparoscopy may cause intraperitoneal adhesions hampering tubo-ovarian relationship and furthermore, may result in adverse pregnancy outcomes such as uterine rupture due to inappropriate repair of the myometrial defect (Tanos and Toney, 2019). The reproductive surgeon should be able also to discuss with the patient, the advantages and disadvantages of performing myomectomy for indications such as unexplained infertility, improving IVF success, and recurrent pregnancy loss (Christopoulos et al., 2017; Metwally et al., 2020). If laparoscopy is contemplated, sufficient expertise with intracorporeal suturing and knot tying is essential to be able to perform a secure layered closure of the myometrial defect. Intraoperatively, it is important to rigorously follow the principles of microsurgery to avoid pelvic adhesions and impairment of the tubo-ovarian relationship that will hamper the chances of spontaneous conception and also conception with IVF.

**Figure 5:**
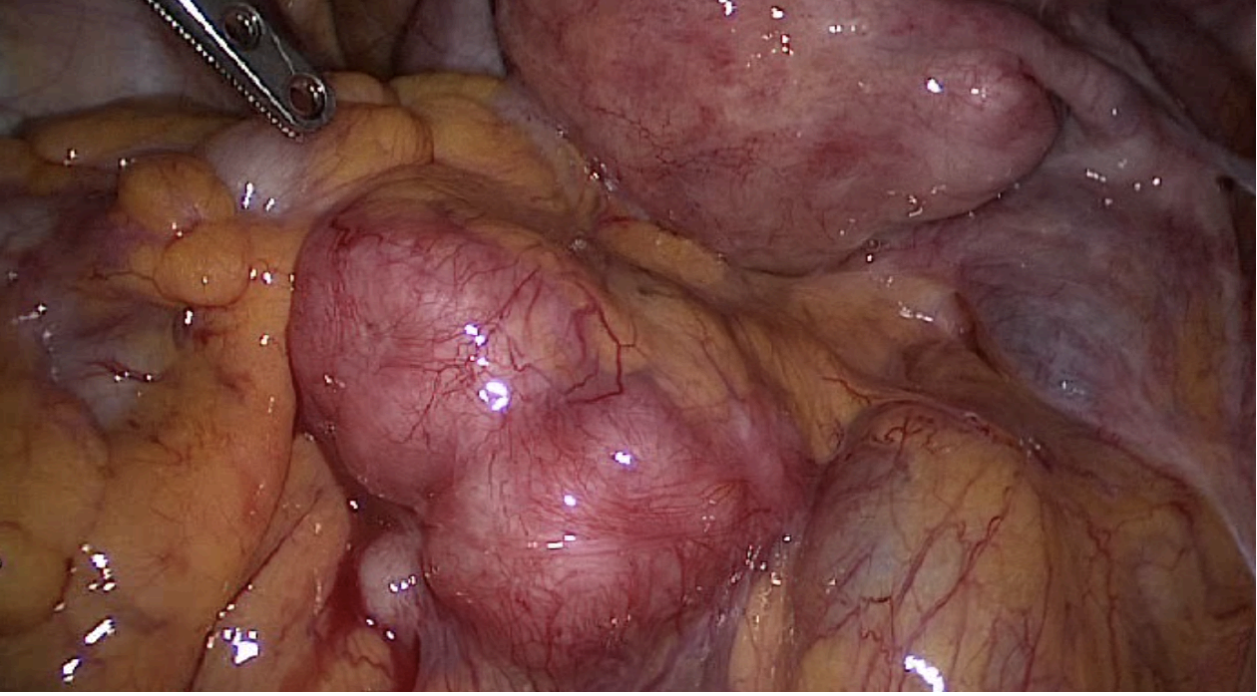
Parasitic myoma embedded in the mesocolon after laparoscopic myomectomy and uncontained power morcellation.

**Figure 6:**
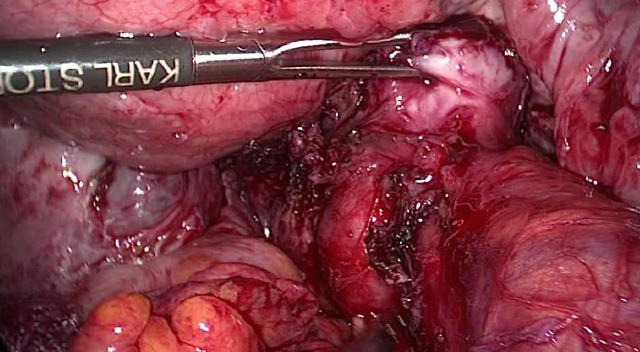
Adenomyoma obstructing the left ureter in a patient who had undergone laparoscopic adenomyomectomy.

**Figure 7:**
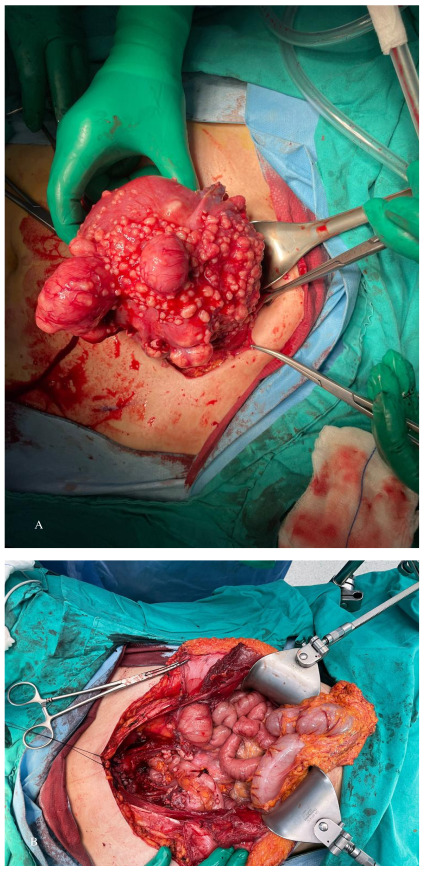
Peritoneal leiomyomatosis in a patient who had undergone robotic myomectomy and uncontained power morcellation 10 years earlier. Appearance of the uterus (a) and the abdominal cavity (b) at laparotomy. a. Uterine serosa covered with parasitic myomas in a patient with peritoneal myomatosis b. Peritoneal myomatosis treated with laparotomy, hysterectomy, omentectomy, and bowel resection.

Another important aspect of laparoscopic myomectomy is the removal of the excised specimen/s from the abdominal cavity (Pepin et al. 2021). Although much emphasis is put on inadvertent morcellation of a leiomyosarcoma, more common is the occurrence of parasitic myomas, disseminated intraperitoneal leiomyomatosis, or adenomyosis when uncontained power morcellation is used (Urman et al., 2016; Boza et al., 2019) (Figures 5,6,7). Alternatives to uncontained power morcellation are morcellation in a bag or removal of the excised tissue through a colpotomy after intraabdominal or vaginal sectioning using a scalpel if required (Aksu et al., 2015). One should be aware of the fact that in order to completely prevent the dissemination of a Stage 1 leiomyosarcoma, myomectomy should be avoided altogether which is practically not possible.

### Surgery for endometriosis

Absolute indications of surgical treatment include pain unresponsive to medical treatment, suspicion of malignancy, and obstructive organ symptoms. Whether and to what extent surgery improves fertility or IVF outcomes is unclear but a number of IVF failures without another explanation may bring about surgery before a subsequent attempt (De Ziegler et al., 2011). While a meta-analysis of available studies suggest that surgical treatment of deep endometriosis may improve ART outcomes, none of the studies was a randomised trial (Casals et al., 2021). Risks must be weighed prior to attempting a complicated procedure such as deep endometriosis surgery.

The endometriosis surgeon should not only have expertise in tackling severely distorted pelvic anatomy but also have a solid grasp on other aspects of the disease such as alternative forms of treatment (medical and IVF), the effect of endometrioma and its removal on ovarian reserve and IVF outcomes, the impact of deep endometriosis on fertility, and fertility preservation options (Chapron et al., 2019). To provide the patient with a holistic approach, endometriosis surgery should be ideally performed preferably by reproductive surgeons who should have a thorough understanding of pelvic spaces, vasculature, and nerves and are experienced in techniques of retroperitoneal dissection.

In most instances surgery is not indicated or may not serve the patients’ best interests and alternatives to surgery should be discussed (Kho et al., 2018). This is most true in women who are asymptomatic, in their earlier reproductive years, and who have not completed their family. Delaying surgery whenever possible will not only prevent future repeat interventions for this chronic and recurring disease but also avoid de-novo pelvic adhesions and will more likely preserve the ovarian reserve (Somigliana et al., 2011; Tandoi et al., 2011). A conservative approach should be adopted in the presence of infertility especially when there are no or only mild pain symptoms.

**Figure 8:**
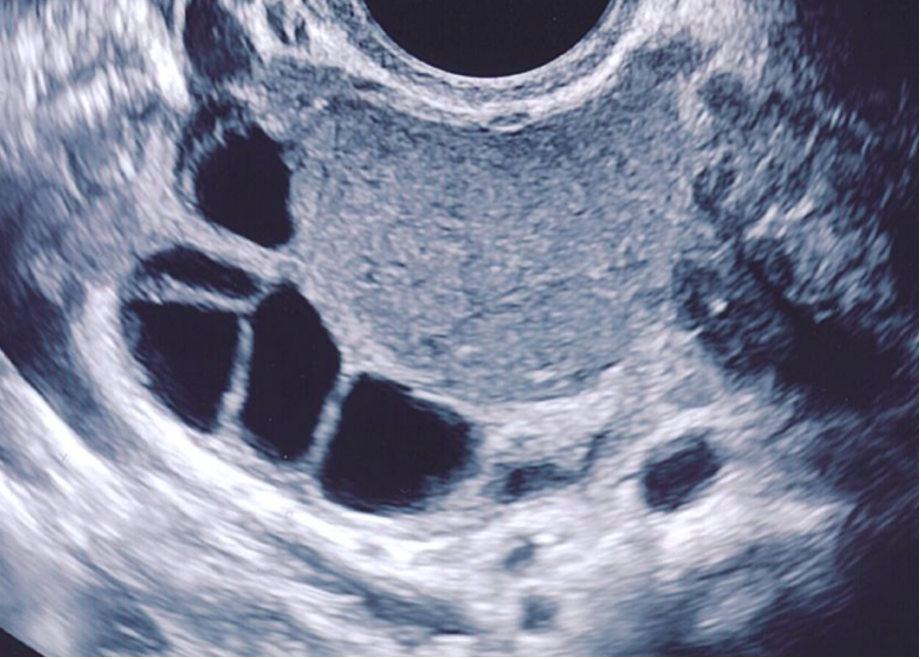
Follicles growing on the periphery of an endometrioma in response to ovarian stimulation.

When viewed through the glasses of a general gynaecologist or a gynaecological surgeon, endometriomas being pathological ovarian cysts should be removed. However, endometriomas do not prevent or decrease ovulation or conception from the affected gonad in the absence of another factor (Leone Roberti Maggiore et al., 2015; Leone Roberti Maggiore et al., 2017). Despite earlier studies indicating over 50% spontaneous conception rates in women with endometriomas subjected to surgical removal by laparoscopy or laparotomy, this is most likely an overestimation as some of the patients included in these studies may not have attempted spontaneous conception prior to surgical intervention and may not have been infertile to start with (Jones and Sutton, 2002; Vercellini et al., 2009). Furthermore, several shortcomings of these studies such as lack of complete follow-up, and most importantly lack of a control group cloud the conclusions that have been reached (Vercellini et al., 2009). As such they do not necessarily represent evidence for a beneficial effect of excision on spontaneous fertility. In patients who have not tried but are planning to have a child, adequate time should be spared for spontaneous conception as approximately half of the patients will do so within 12 months when there are no additional infertility factors (Leone Roberti Maggiore et al., 2015; Leone Roberti Maggiore et al., 2017). IVF should be the second-line approach. Endometriomas do not appear to increase in size during ovarian stimulation for IVF nor hamper the outcome of stimulation; whereas surgery will almost always diminish ovarian reserve and adversely affect IVF outcomes at least in regard to cumulative pregnancy rates (Seyhan et al., 2015). When the oocyte yield from an ovary with an in situ endometrioma is compared with the oocyte yield from an ovary without an endometrioma the difference between the mean number of oocytes retrieved was statistically significant but clinically negligible at less than one oocyte (mean difference -0.23 oocytes, 95% confidence interval; -0.37 to -0.10) (Hamdan et al., 2015). Often, one can see growing follicles in response to gonadotropin stimulation in patients with endometrioma and no visible antral follicles on basal ultrasound examination (Figure 8). While in situ endometriomas can render follicle aspiration incomplete and decrease the total number of oocytes collected, they do not seem to affect oocyte developmental potential or clinical pregnancy rates per stimulation and their removal is not mandatory before an IVF cycle (Benaglia et al., 2018; Alshehre et al., 2021).

More often than not, careful consideration is not given to the preoperative ovarian reserve and future fertility prospects. Careful ultrasonographic assessment of the pelvis will reveal patients at risk for malignant cysts and also deep endometriosis lesions that are commonly associated with endometriomas (Alson et al., 2022). The surgical technique of endometrioma removal requires strict adherence to delicate microsurgical principles of tissue handling. Undue traction of the cyst capsule and subsequent bipolar coagulation of the resulting bleeders should be avoided. The surgeon should be aware of the tissue destruction caused by different energy modalities and the availability of alternatives such as locally applied haemostatic agents and sutures (Ata et al., 2015). Complete eradication of pathology should be the ultimate goal, however, it will be useless and more frustrating to deal with for the patient, if associated also with complete eradication of ovarian function (Koninckx et al., 2021). Ablation of the pseudocyst wall with plasmajet or sclerotherapy, both without stripping may be less detrimental to ovarian reserve but more studies are needed (Roman et al., 2014; De Cicco Nardone et al., 2020; Crestani et al., 2023; Daniilidis et al., 2023).

**Figure 9:**
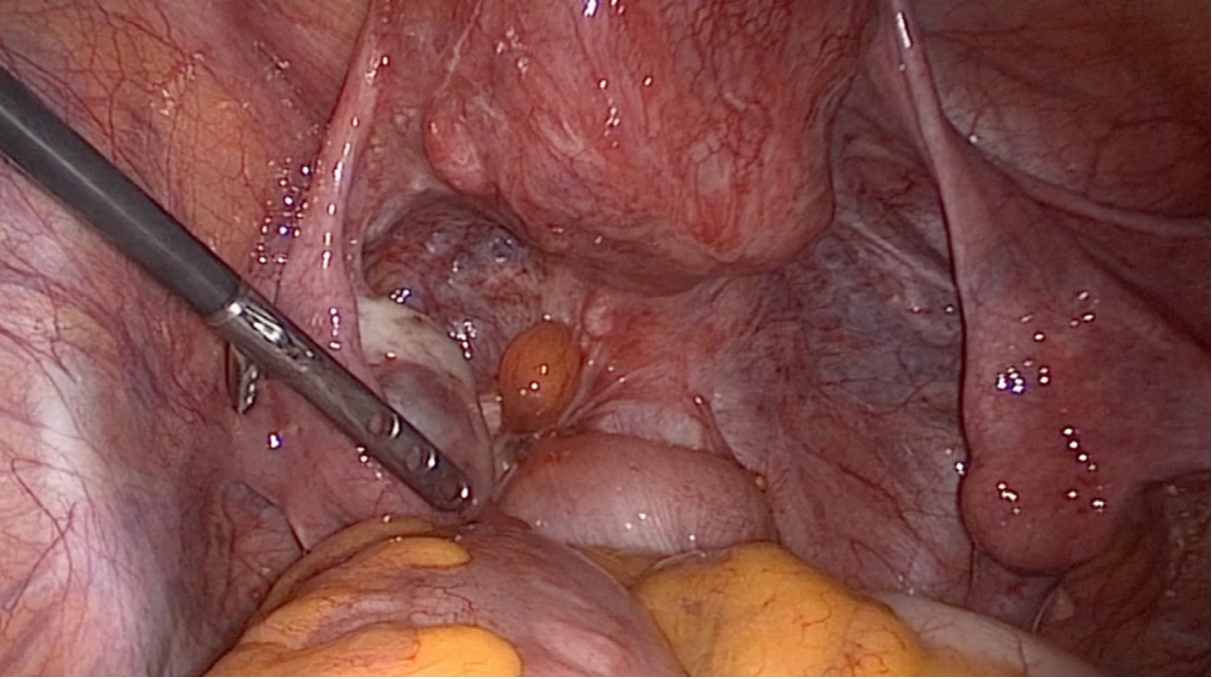
Infertile woman with severe pelvic pain, rectal endometriotic nodule and adenomyosis.

Deep endometriosis when symptomatic may necessitate surgery. Surgery often entails extensive dissection of the pelvis and quite commonly together with bowel resection, leaving behind large areas of exposed peritoneal surfaces that may lead to severe adhesions. Deep endometriosis lesions are frequently associated with ovarian endometriomas and adenomyosis further complicating the treatment and decreasing postoperative pregnancy rates be it spontaneous or with IVF (Exacoustos et al., 2014; Higgins et al., 2021; Squillace et al., 2021; Sudhakar et al., 2022). Isolated nodules without peritoneal and ovarian endometriosis are rare, however, they are commonly seen together with adenomyosis (Figure 9). Gains from surgery such as relief of pain may be offset by reduction of ovarian reserve and complications of surgery such as bowel and urinary dysfunctions (Byrne et al., 2018). It is still uncertain whether deep endometriosis surgery with or without bowel resection improves fertility in the infertile patient (Iversen et al., 2017; Daniilidis et al., 2022; Raos et al., 2023). In patients contemplating pregnancy, careful assessment of all pelvic compartments, the uterus (presence or absence of adenomyosis), the ovaries (presence or absence of ovarian endometriomas and measurement of ovarian reserve), and patency of the fallopian tubes is necessary. Some patients may be better served by initial treatment with IVF or oocyte/embryo banking followed by surgery prior to embryo transfer. It should also be noted that surgery for severe endometriosis is not complication-free, and some rare complications like bowel or urinary fistula may delay the treatment of infertility. Such could be regarded as another reason not to prioritise surgical treatment for infertile endometriosis patients in the absence of an absolute indication.

The notion of fertility preservation has been strongly emphasised recently due to the growing body of evidence relating surgery with adverse fertility outcomes such as diminished ovarian reserve, de-novo pelvic adhesions resulting in blocked tubes, and recurrence of endometriosis resulting in repeated surgical interventions (Latif et al., 2022; Elizur et al., 2023). Accordingly, the patient should be counselled regarding the possibility and outcomes of oocyte and embryo cryopreservation prior to embarking upon surgery. Patients with an indication for surgery (severe pain, hydrosalpinx, and obstructive bowel symptoms) are possibly best served with prior fertility preservation through oocyte/embryo banking. Cobo et al. recently reported that 43% of 1,044 women who had frozen oocytes due to endometriosis returned to use their oocytes (Cobo et al., 2020). These figures highlight the necessity of fertility preservation for women with endometriosis (Somigliana and Vercellini, 2020).

The choice of surgery or IVF as the initial management of endometriosis-associated infertility should not be biased by the attending surgeon being more skilled in one than the other. A reproductive surgeon who is skilled in both IVF and reproductive surgery, as opposed to a gynaecologic surgeon without infertility expertise, is likely the best person to provide impartial counselling with a holistic view.

### Surgery for adenomyosis

**Figure 10:**
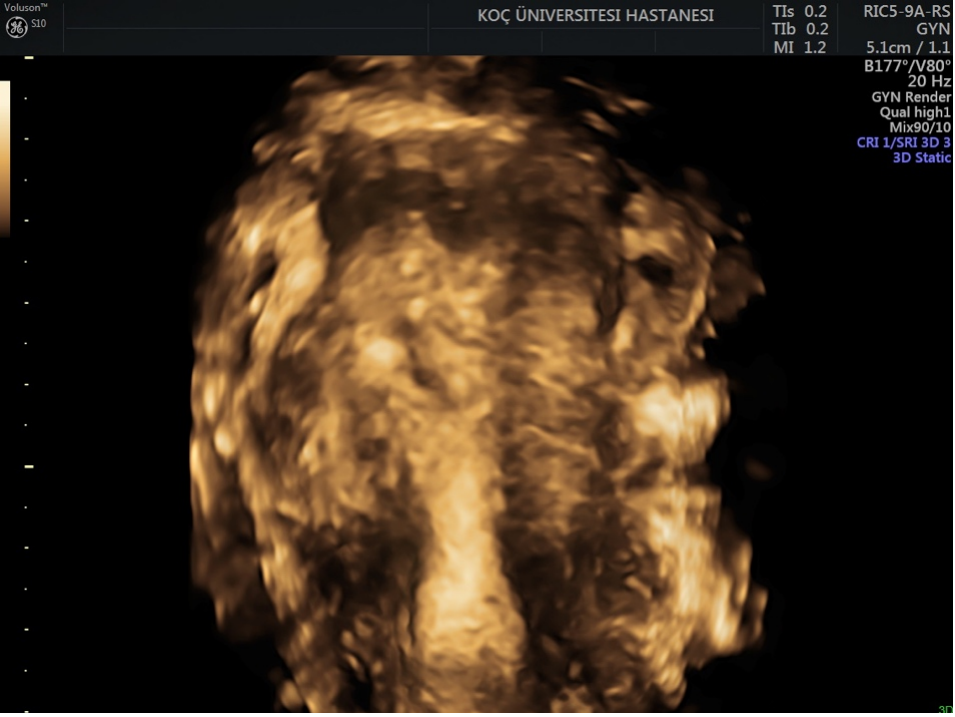
Postoperative 3D ultrasound image of a patient who underwent laparoscopic adenomyomectomy erroneously diagnosed as fibroid before surgery. Note the large myometrial defect on the right cornual portion and irregular endometrial cavity with multiple adhesions. Also, note the enlarged and irregular junctional zone on the fundal region suggesting presence of adenomyosis.

**Figure 11:**
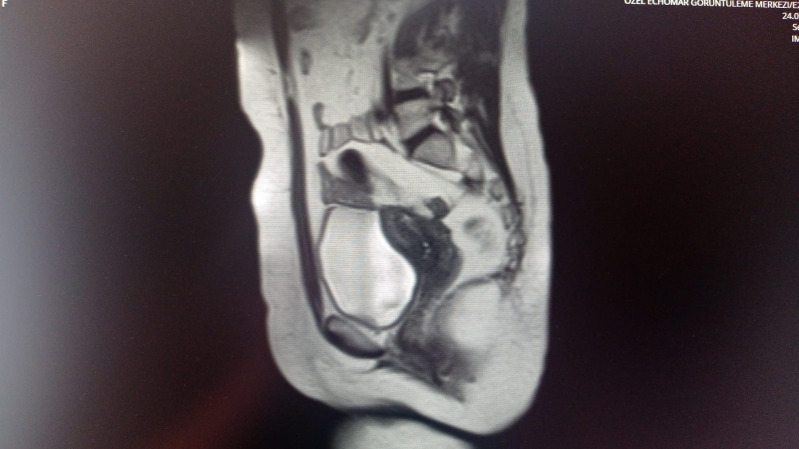
Postoperative magnetic resonance image of a patient who underwent laparoscopic adenomyomectomy resulting in a huge fundoposterior myometrial defect precluding pregnancy.

While the benefit of surgical excision of adenomyosis is uncertain, the complexity of it is crystal clear. The use of pelvic imaging has demonstrated the existence of different forms of adenomyosis, notably allowing distinction between lesions of the external myometrium and those of the internal myometrium (Bourdon et al., 2021). Moreover, the ultrasonographic evaluation of the type and extension of adenomyosis in the myometrium seems to be important in correlation to the severity of symptoms and infertility (Exacoustos et al., 2020). While some forms of the disease are commonly associated with deep endometriotic lesions others may be isolated within the uterus (Bourdon et al., 2021). Internal adenomyosis (disruption of the junctional zone by mutated endometrium and infiltration into the myometrium) and external adenomyosis (invagination of the retrocervical deep lesions into the neighbouring myometrium of the posterior lower uterine segment) should be dealt with separate treatment algorithms depending on the patients’ symptoms, presence or absence of infertility, whether IVF treatment is contemplated and whether IVF treatment has failed in the absence of other obvious reasons (Dueholm, 2017; Horton et al., 2019). Sometimes the disease may be difficult to differentiate from myomas and this is especially true for focal lesions referred to as adenomyomas. Removal of adenomyomas is challenging compared to myomas as the former do not have a pseudo capsule that facilitates dissection. As for diffuse disease, the borders between adenomyotic myometrium and eutopic endometrium are always blurred as are the borders between adenomyotic and normal myometrium (Huang et al., 2015). Once an incision is made into adenomyosis, it is difficult to decide when and where to stop the excision and how to close the resulting myometrial defect. Ambitious resection of adenomyosis, akin to oncological surgery, almost always risks breach of the endometrial cavity with subsequent adhesion formation, and loss of too much myometrial tissue, which can cause irreparable damage that can endanger a future pregnancy due to risk of uterine rupture. A reproductive surgeon would be the best person to decide when the risks of adenomyosis excision in an infertile woman are worth its possible benefits. If surgery is undertaken, a reproductive surgeon would be the one most likely to balance how much disease can be left in situ while maintaining a functional uterus, as opposed to surgeons with oncologic background, who tend to prioritise maximal excision above all. Figures 10 and 11 show postoperative images of two infertile patients who underwent laparoscopic adenomyomectomy by two different gynaecological oncologic surgeons. The patient with the lateral wall defect preoperatively was misdiagnosed as having a fibroid, and the recognition of the error did not stop the surgeons from attempting to remove the entire lesion at the cost of breaching the endometrial cavity and loss of almost half of the muscle mass on that side. The patient had undergone hysteroscopic adhesiolysis three times with incomplete resolution by the same team. Unfortunately, we had to advise against pregnancy by any means to both patients, due to the risk of uterine rupture. They represent extreme examples of the problem we described above.

Surgery for adenomyosis entails the removal of focal (adenomyoma) or diffuse disease. In the reproductive-aged women surgical treatment may be considered in the presence of severe pain, abnormal uterine bleeding unresponsive to conservative measures, otherwise unexplained recurrent pregnancy losses, or failed IVF attempts (Tan et al., 2018; Younes and Tulandi, 2018). A systematic review showed that excision of adenomyosis is effective for symptom control such as menorrhagia and dysmenorrhea and most probably for adenomyosis-related infertility. For preserving fertility and relieving symptoms, medical treatment is usually the first choice, whereas excisional surgery could be performed for refractory adenomyosis. Patients with focal adenomyosis appeared to have higher pregnancy rates after conservative surgery compared with diffuse adenomyosis, whereas a higher incidence of uterine rupture was reported after surgery for diffuse adenomyosis (Tan et al. 2018). Removal of adenomyomas indenting the uterine cavity is more difficult as the endometrium is likely to be breached. The residual myometrial defect may also be substantial after removal of large adenomyomas. Laparotomy as opposed to laparoscopy should be considered in these patients and the uterine muscle flap method can be preferred to prevent large myometrial defects (Kwack et al., 2018; Osada, 2018; Zhu et al., 2019). Surgery for diffuse adenomyosis should be performed only after exhaustion of all other treatment options.

Internal adenomyosis is an extension of endometrial glands and stroma into the adjacent junctional zone and the inner myometrium and is most probably responsible for abnormal uterine bleeding, infertility, and implantation failure while external adenomyosis is the invasion of the outer muscular layer by deep infiltrating peritoneal lesions (Bourdon et al., 2021). Careful consideration should be given to the assessment of the uterine cavity linking ultrasound findings with those at hysteroscopy to understand whether the endometrium is conducive to implantation (Gordts et al., 2018).

### Surgery for caesarean scar defects

Caesarean scar defect, uterine niche or isthmocele is an iatrogenic complication of caesarean section that has attracted widespread attention due to its association with menstrual disturbances and infertility (Tulandi and Cohen, 2016; Murji et al., 2022). A niche was defined as an indentation at the site of the uterine incision performed for a hysterotomy with a depth of at least 2 mm. Basic measurements include niche length and depth, residual and adjacent myometrial thickness in the sagittal plane (Jordans et al., 2019). According to a recent study utilsing these criteria, the incidence of a uterine niche was significantly higher in women who had an elective (20/45; 44.4%) compared with those who had an emergent (21/115; 18.3%) caesarean delivery (Feldman et al., 2022). Special emphasis has also been put on the closure of the uterine incision (single versus double layer, locking versus non-locking, incorporation of the decidua into the suture versus not, suturing of the visceral and parietal peritoneum versus not) (Sholapurkar, 2018). However, a large randomised controlled trial including 1144 and 1148 women who underwent caesarean with a single or double layer closure, respectively, did not report differences between live birth rates, pregnancy rate, need for fertility treatments, mode of delivery, or uterine ruptures in subsequent pregnancies (Verberkt et al., 2023). Two other randomised trials from different centres, with smaller sample sizes, reported similar results (Yilmaz Baran et al., 2021; Yildiz and Timur, 2023). While a systematic review including randomised and non-randomised studies categorically supported double-layer closure based on residual myometrial thickness, they did not report pregnancy rates or fertility status (Genovese et al., 2023).

The reproductive surgeon should be the one who manages symptomatic caesarean scar defects as treatment options should balance the desires of the patient regarding future fertility, the success of the various reported procedures, intraoperative complications, and pregnancy outcomes (Lawrenz et al., 2020). In women who have completed their family and who do not desire another pregnancy, placement of an LNG IUD appears to be the best option (Tower and Frishman, 2013; Armstrong et al., 2023). In women who are interested in becoming pregnant, options include vaginal, hysteroscopic, laparoscopic, or laparotomic correction of the defect. Unfortunately, these treatments have not been compared head-to-head in randomised trials. Only one study compared pregnancy rates in women treated by hysteroscopy versus those who were managed expectantly (Abdou and Ammar, 2018). Hysteroscopic treatment resulted in superior pregnancy rates compared with expectant management (75 versus 32.1%). Compared with vaginal surgery, hysteroscopic resection has been associated with more satisfactory outcomes in terms of intraoperative blood loss, operation time, and hospital stay (Yuan et al., 2022). However, there were no differences in terms of scar reduction and menstrual improvement. Hysteroscopy appears to be the safest procedure in terms of complications. However, this may be due to selection bias as patients with smaller defects and fewer symptoms may have been treated by hysteroscopy. Pregnancy rates cannot be compared due to a small number of patients included in the other treatment groups (Harjee et al. 2021). It may be concluded derived from the limited evidence in the literature that surgical treatment is more effective than expectant management in patients who desire to become pregnant, and hysteroscopy should be the initial approach. Whether the size of the defect and residual myometrial thickness governs the treatment choice should be evaluated in further clinical trials. While a systematic review from 2020 suggested that a laparoscopic or vaginal approach can be preferred for patients desiring future pregnancy as the latter are associated with a thicker residual myometrium, this recommendation is subjective as the authors point out in the paper and effect of different surgical approaches on fertility and obstetric complications are not reported in the original studies (Vitale et al. 2020).

### Surgery for intracavitary uterine lesions

Intracavitary lesions of the uterus, are commonly associated with infertility. These may be congenital (Mullerian anomalies) or acquired (polyps, myomas, intrauterine adhesions, retained products of conception and adenomyosis).

Congenital abnormalities such as septum, besides causing infertility may also result in adverse pregnancy outcomes (Rackow and Arici, 2007; Valle and Ekpo, 2013). Diagnosis has been simplified after the introduction of high-resolution 2D and 3D ultrasound and rarely is it necessary to perform a laparoscopy together with a hysteroscopy (Imboden et al., 2014; Ludwin and Ludwin, 2015). Despite the scarcity of well-designed studies hysteroscopic incision of a septate uterus is a commonly performed surgical intervention. Until recently, this treatment has been assessed only in uncontrolled studies, that suggested a positive effect on pregnancy rates and pregnancy outcomes. However, these studies were biased due to the fact that the participants served as their own controls (Kowalik et al., 2011). In a recent randomised multicentre study, women with a septate uterus and a history of subfertility, pregnancy loss, or preterm birth, were randomly allocated to septum resection or expectant management. The primary outcome was conception leading to live birth within 12 months after randomisation. Despite having important limitations, the study did not show any difference in reproductive outcomes for women undergoing hysteroscopic metroplasty versus controls (Rikken et al., 2021). However, not all women with a septate uterus present with similar reproductive histories. Women with high order pregnancy losses or previous implantation failures pose a special challenge (Tomazevic et al., 2010). A recent systematic review, including studies with different designs, concluded that the septum is associated with higher risk of pregnancy loss and septum incision may decrease the risk of pregnancy losses, however, we think more high-quality studies are needed (Noventa et al., 2022). Given the fact that in experienced hands, hysteroscopic septum incision is a relatively simple and safe procedure, many women are still likely to benefit from such an intervention (Valle and Ekpo, 2013; Alonso Pacheco et al., 2020). As with any reproductive surgery, a hysteroscopic incision of a uterine septum should be undertaken by an experienced reproductive surgeon, who is well- versed in the relevant literature and can provide extensive counselling regarding its pros and cons.

There is only one randomised study showing the effectiveness of hysteroscopic polypectomy in women who were planned to undergo intrauterine insemination (IUI) (Perez-Medina et al., 2005). The study showed statistically increased pregnancy rates in women who had their polyps removed compared to controls. 29% of women in the polypectomy group, compared to 3% in the diagnostic hysteroscopy group became pregnant in the three-month period after the hysteroscopy before the treatment with gonadotropin and IUI was started. In women who started gonadotropin and IUI treatment, the pregnancy rates per woman were 49% and 26% in the intervention and control group respectively. It is commonly accepted that endometrial polyps > 1 cm should be removed in women with infertility or women who are scheduled to undergo IVF treatment (Bosteels et al., 2010). Preference should be given to office hysteroscopy as this is easier to perform and rarely necessitates general anaesthesia.

Prioritising medical termination of pregnancy over surgical interventions has recently gained momentum to obviate the complications associated with curettage such as uterine perforation and formation of intrauterine adhesions both of which may adversely affect reproductive performance. In patients with retained products of gestation (RPOC) consideration should be given to expectant management as the outcome did not differ in patients who were randomised to waiting for 8 weeks versus medical treatment (Tzur et al., 2022). If RPOC are not expelled hysteroscopic resection should be preferred over ultrasound-guided or blind curettage (Meshaal et al., 2022). Indeed, the latter should ideally be avoided to the possible extent. Office hysteroscopy can be considered when the RPOC is < 2 cm in diameter (Mohr-Sasson et al., 2022). The application of intrauterine barrier gels should be considered to prevent intrauterine adhesions due to increased risk of this complication after surgery under a hypoestrogenic environment.

### Uterine transplantation surgery

Uterine transplantation is the new frontier in reproductive surgery. Not only it opens up a new avenue to the possibility of motherhood in women who do not have uterus or a functional endometrium, but as recently suggested it may also be employed in transgender women (Brannstrom et al., 2021; Johannesson et al., 2022; Richards et al., 2023). The uterus can be harvested from cadavers or living donors with success rates reported as 64 and 78% respectively and yielding cumulative live birth rates exceeding 80% (Brannstrom et al., 2021; Brannstrom et al., 2023). The surgery is difficult and technically challenging. High intra and postoperative complication rates, the need for long-term immunosuppressive treatment to maintain the transplant, and increased risk of preterm labour and delivery with its associated costs currently limit the uptake of the procedure. Refinement of laparoscopic techniques may result in the application of minimally invasive approaches that may ease the burden of surgery for the donor and the recipient alike (Puntambekar et al., 2018; Puntambekar et al., 2019).

## Conclusions

Taking into consideration the rapid pace of advances in medical practice in general and reproductive medicine and surgery in particular, it is more important than ever that surgical procedures that may impact fertility in women who plan to conceive in the future should be in the armamentarium of trained reproductive surgeons. Reproductive surgery is all about retaining organs with better function after surgery than before surgery. As such, it is much more than opening blocked tubes. Besides being an excellent technician, the reproductive surgeon must also be sufficiently equipped with knowledge concerning ovarian, tubal, and uterine physiology, reproductive endocrinology, fertility preservation, ART, and gynaecological imaging techniques. While ART should not directly replace reproductive surgery when the latter presents a viable option for even multiple spontaneous pregnancies in the future, also the availability of ART as a backup procedure should not lead to a less careful and relaxed attitude towards surgery. Treatment of gynaecological cancers by trained gynaecologic oncologists will benefit the patients in terms of higher disease-free survival and cure rates (Mayer et al., 1992; Bilimoria et al., 2009). A more recent study showed that women with endometrial intraepithelial neoplasia may benefit from surgery with a gynaecologic oncologist rather than a general gynaecologist to reduce costs and adverse events associated with a second surgery (Chaiken et al., 2022). Maternal- foetal medicine specialists will miss less of the abnormal markers of trisomies compared to general obstetricians (Blessed et al., 2001; Agathokleous et al., 2013). Just like the above, infertile women or women who contemplate childbearing in the future will be best served if reproductive surgery is undertaken by or under the supervision of trained reproductive surgeons. We have reviewed several surgical conditions that are often encountered during the care of infertile women. The aim was not to provide a description of the ideal surgical approach to each but rather to highlight the gaps in knowledge and numerous controversies surrounding each of them while summarising the current opinion on their management. As such, the work clearly demonstrates the need for in-depth and up-to- date knowledge of the management of infertility to provide a holistic approach to patients. This is only possible by focusing one’s mind and practice in the field of reproductive medicine and surgery, which is certainly unattainable while practicing in multiple fields. So, infertile patients will be best served by reproductive surgeons and every reproductive medicine team should have at least one expert reproductive surgeon. The time has come.
